# Measuring equality in access to urban parks: A big data analysis from Chengdu

**DOI:** 10.3389/fpubh.2022.1022666

**Published:** 2022-10-06

**Authors:** Weiwei Dai, Suyang Yuan, Yangyang Liu, Dan Peng, Shaofei Niu

**Affiliations:** ^1^College of Architecture and Environment, Sichuan University, Chengdu, China; ^2^Park City Center, China Southwest Geotechnical Investigation and Design Institute Co., Ltd., Chengdu, China; ^3^Research Center of Urban Renewal, Sichuan Provincial Architectural Design and Research Institute Co., Ltd., Chengdu, China; ^4^Urban Development Innovation Studio, China Southwest Geotechnical Investigation and Design Institute Co., Ltd., Chengdu, China

**Keywords:** urban parks, accessibility, 2SFCA, equality in access, big data, machine learning

## Abstract

Spatial equality of parks is a significant issue in environmental justice studies. In cities with high-density development and limited land resources, this study uses a supply-demand adjusted two-step floating catchment area model (2SFCA), paying attention to residents' subjective preferences and psychological accessibility. It assesses equality of access to urban parks from two dimensions: spatial equality and quantitative equality at a fine scale of 100 × 100 m grid resolution. The spatial equality of urban parks in Chengdu is measured under different transportation modes (walking, cycling, and driving) based on multi-source geospatial big data and machine learning approaches. The results show: (1) There were significant differences in the spatial distribution of park accessibility under different modes of transportation. The spatial distribution under walking was significantly influenced by the park itself, while the distribution of rivers significantly influenced the spatial distribution under cycling and driving; (2) Accessibility to urban parks was almost universally equal in terms of driving, relatively equal in terms of cycling, and seriously unequal in terms of walking; (3) Spatial local autocorrelation analysis shows that park accessibility tended to be significantly clustered, with little spatial variation; and (4) The supply and demand of urban parks were relatively equal. The results can help urban planners to formulate effective strategies to alleviate spatial inequality more reasonably and precisely. The applied research methods can further improve the system of scientific evaluation from a new perspective.

## Introduction

Urban parks are part of public green infrastructure that provides ecological, health, social, and economic benefits for the urban environment and residents (1–4). However, global urbanization, driven by population growth, is experiencing faster growth and is expected to rise to 68% by 2050 (5). Urbanization has put significant pressure on the environment, such as increasingly congested and polluted cities, parks in short supply, and severe problems of environmental or green gentrification (6, 7). Sustainable green environments cluster in wealthy, predominantly white neighborhoods, far from poor, predominantly communities of color, becoming an added economic value to real estate (7). A good supply of urban parks can bring significant benefits to people, and the challenge of urbanization may also provide an important opportunity for sustainable urban management.

At present, decision-makers or planners are still accustomed to using indicators that can only roughly summarize the overall level of urban park construction. These indicators are the green coverage rate, green space rate, and park area per capita. However, they often ignore the differences in the spatial distribution of public resources (8–10). When the supply of resources is disproportionate to the demand, there will be “inequality” in spatial distribution, bringing about a series of social contradictions. The essential elements of the built environment and basic public services provided by the government should guarantee equality in access and allocation of urban parks as a human right to meet the needs of different social groups (11–13). Availability and spatial distribution of public service resources are important factors for evaluating equality (14). An accessibility evaluation based on the balance of supply and demand helps to understand whether urban parks provide sufficient quantity and equality.

In Europe and the United States, there are more studies on parks' green space equality, but there is a lack of research in China. China has a large population and is the fastest-growing developing country. As China's economy transforms, social inequalities and the widening gap between the rich and the poor are magnified and reflected in the residential space distribution. It is unclear whether the resources of parks and green spaces are equitably distributed and whether they can continue to meet the needs of residents (15, 16). The limited evidence currently available suggests that access to urban parks in China's megacities is deteriorating (17). As a mega city in Southwest China, Chengdu's urban development is similar to that of most Southeast Asian countries and practices the new development concept of the “Park City” demonstration area. At this moment, the park accessibility study can help optimize equality-oriented park planning, as well as provide decision-making guidelines for improving the city's livability and healthy and sustainable development from the perspective of overall synergistic urban development.

### Green justice and access to the park

As a social movement, environmental justice mainly originated in the United States (18). It can be explained from three different dimensions, including distributive justice, procedural justice, and interactive justice (19). Among them, distributive justice emphasizes the equitable distribution of environmental resources use rights and protection obligations, which is the main focus of the present study (20). From the perspective of sustainable urban development, green space is an important research object of environmental justice (21). In the past 20 years, the inequitable accessibility of public green spaces has attracted the attention of scholars at home and abroad (22) (23). This study defines green justice that only emphasizes the justice of green spaces in urban and rural areas. It is a branch of environmental justice (24). The essential objectives of spatial planning and relevant policies are to improve the accessibility of urban green spaces and to ensure the fair rights of social groups in different spaces of the city to use green spaces in order to maximize its environmental, social, and health benefits.

Dai (22) studied the impact of different races and economic and social backgrounds on the accessibility of parks and proved the relationship between these factors and park accessibility. The scale of study significantly impacted indicators of park provision, and it was easier to identify the causes of inequality at small scales (25, 26). Xing (27) explored whether park accessibility is equal when taking teenagers as a research object.

### Factors influencing the accessibility of urban parks

Park accessibility is influenced not only by its size but also by other properties, such as its functions, types, landscape quality, equipment, maintenance, and the psychological perception of the public (28–31). There are many factors that affect accessibility, but they can be summarized in three directions: the supply side (urban parks), the demand side (users), and the path (connecting the supply and demand). Currently, there are few studies that consider the subjective psychological level of demanders. Park (32) tried to explore the factors that affect people's psychological level of accessibility and found that the richness of the park's content, the condition of the park's surroundings, and the users' psychological tolerance for the distance traveled are the three main factors affecting psychological accessibility. Dony et al. (33) introduced the factor of park facilities (e.g., basketball courts, badminton courts, etc.) to evaluate the effect of park attractiveness on accessibility. Some scholars believe that perceived accessibility is more important than geographical access (34). Finally, traditional quantitative modes ignore non-physical variables, such as safety, culture, personal preferences, or motivations (31).

### A method of evaluating the accessibility of urban parks

The models and methods used to evaluate park accessibility are constantly being innovated. Among them, survey research (35), statistical index calculation (36), buffer analysis (37), and distance to the nearest park (38) are simple and easy to operate methods. However, they all ignore the actual traffic network and have significant calculation errors. The cost-weighted distance method (39) considers the actual road, but the setting of some indices is subjective. The gravity-based model (8) comprehensively considers the attraction factor of the park, but the calculation is complicated. The network analysis method (40) also has complex operations and high requirements for data sets that are difficult to obtain. Space syntax (41) considers human behaviors and activities, so it is more accurate to analyze the potential use of space. It is easy to operate and easy to compare results. Nevertheless, it does not consider the attractiveness of the park. The two-step floating catchment area method (2SFCA) (42) overcomes the limitations of administrative boundaries and considers factors affecting accessibility from the supply and demand dimensions, making it more comprehensive. Additionally, this method performs quantitative spatial analysis based on the ArcGIS platform, and the results can be visualized.

Scholars began to use new technology to improve the accuracy and efficiency of various methods. Zheng et al. (25) used an open urban big data platform to calculate park accessibility by invoking a map Application Program Interface (API) to obtain more accurate housing and path information. Some scholars have used the 2SFCA method to evaluate park accessibility using mobile phone data (43, 44).

### Improvement of the 2SFCA method

Although the traditional 2SFCA method evaluates accessibility from supply and demand sides, there are certain limitations. Researchers continue to optimize the four aspects of the distance decay function, search radius, travel mode, and demand or supply competition model for more accurate accessibility results.

First, researchers have introduced a distance decay function to reduce the resulting error caused by using straight-line distance. E2SFCA introduces an attenuation function with a section of stepped jump decay, which is a more realistic response to the influence of distance decay on accessibility, but the weight setting is more subjective (45). Ga2SFCA introduces Gaussian function, and the decay rate of accessibility increases first and then slows down with increasing distance, and is currently widely used (46).

Second, some researchers made improvements to the search radius after considering that suppliers and demanders may have different supply capacities and demands due to their conditions. V2SFCA can adjust the search radius to cover a sufficient scale of supply and demand but is highly subjective. NN2SFCA considers the option of the demanders but assumes that the demanders will only choose the nearest facility within the threshold range, which does not fully match the real life (47).

Third, a few studies consider multiple traffic modes. CB2SFCA considers the impact of different commuting behaviors on the accessibility of daycare centers (48). However, this method requires a large amount of fine-grained residential travel data, which is difficult to obtain and generalize.

Finally, researchers have considered different attractions of supplier and different needs of demanders. O2SFCA considers competition between demanders, but this method requires good data and it is difficult to promote in practice (49). 3SFCA considers competition between multiple supply points (50), and i2SFCA introduces the huff model to measure the potential saturation of a single facility (51). However, the above methods are only unilaterally improved from the supply or demand side.

### Research gap and purpose

There are four research gaps in the literature. (1) There is a lack of comprehensive methods, which can improve the distance decay function, search radius, travel mode, and demand or supply at the same time, based on the application of big data and new technologies in order to measure equality in access to urban parks at large scale. (2) More understanding of people's subjective preferences and psychological accessibility from a people-centered perspective is lacking. (3) Few studies have considered the impact of urban environmental interventions around parks on equality in access to urban parks. (4) There is a lack of evidence to assess equality in access to urban parks based on fine-grained grid resolution.

**Figure 1:**
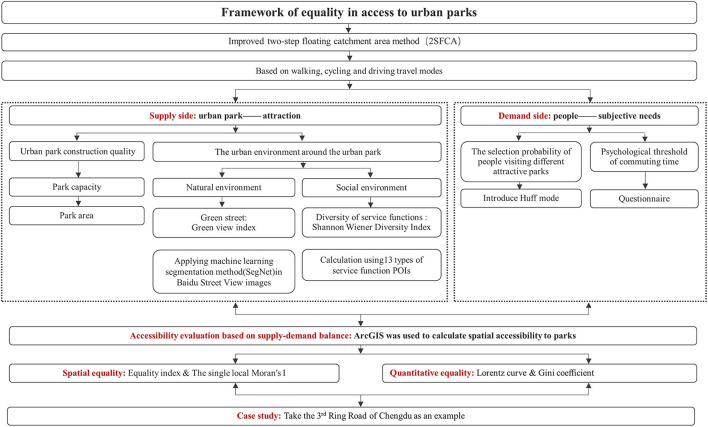
Research framework.

In Figure 1, in this study, the spatial accessibility of parks with different supply levels is first calculated based on the traditional 2SFCA method under multiple transportation modes (walking, cycling, and driving) at a fine scale. The impacts of the natural and social environments around the urban parks on the attractiveness of the parks as well as people's subjective right to choose to visit different parks were considered. Then, the equality index of park accessibility is calculated, and the spatial equality of the parks is studied by combining the Lorenz curve and the Gini coefficient. The spatial autocorrelation analysis is performed using the local Moran's I index. Finally, the spatial disparity in people's accessibility to urban parks within the Third Ring Road in Chengdu, China, was evaluated. This paper can help answer the following three questions: (1) For a central city, how to evaluate equality in access to urban parks on a more realistic, comprehensive, and accurate scale from people's perspectives? (2) What are the main problems with equality in access to urban parks within the Third Ring Road in Chengdu? and (3) What policy implications can be proposed based on our findings in order to serve not only Chengdu but other cities worldwide?

The remainder of this paper is organized as follows. Section 3 gives an overview of the traditional 2SFCA and details how to improve 2SFCA and calculation methods of other critical indicators in this paper. Section Discussion describes a case study of urban parks within the Third Ring Road of Chengdu using the evaluation framework constructed in this study. Section Conclusion presents the results of a comparative analysis of different areas under different modes of transportation and further identifies areas of inequality in access to urban parks. Section Funding draws out the theoretical and policy implications of this research.

## Materials and methods

### Improved 2SFCA method

The calculation of supply and demand coefficients was improved based on the traditional 2SFCA method in order to evaluate spatial access to urban parks. For supply improvement, the calculation of attraction coefficient Sj not only considered the park area in the traditional 2SFCA, but also considered the park's urban environment. The street green view index and variety of service functions reflect the urban natural and social environment around the park. For demand improvement, the demand coefficient Pi added consideration of the probability of selection (Probi) of people by incorporating the Huff model.

Many visitors avoid parks if they believe that the cost of travel outweighs any benefit they receive from the park. The park's attraction also determines whether people are willing to overcome all kinds of resistance to visit the park. Many factors affect the attractiveness of parks, such as the size of the parks and the number of amenities (33). This study will consider the impact of the external urban environment around the park on the attractiveness of the park from a macroscopic and holistic perspective of the city. In terms of internal park construction, due to the large number of subjects in this study, it is not possible to finely assess the quality of the internal construction of each park, so only indicators of the park area are introduced Regarding the external park environment, the diversity of the service function and street green view index in the park service area were introduced to investigate the influence of the natural and social environment in the city on the park's attractiveness.

The park area is the most essential factor that reflects the attractiveness of the park in the original formula of the 2SFCA method. On the one hand, it reflects the rank of the park, and generally the higher the rank, the larger the park's area; on the other hand, it directly determines the park's capacity.

Green streets are an essential aspect of connecting parks. The green street is both a new green activity site and a good commuting environment for park visitors. Considering parks and green street spaces can ease the problems of green gentrification in core cities where usable land is scarce (23). The street green view index is used in this study to assess the green street spatial situation (52, 53). We use a deep learning-based image segmentation algorithm (Pyramid Scene Parsing Network, PSP-Net) to measure the density of categorized environmental factors in Baidu Street View panoramas, such as percentages of vegetation, sky, and buildings. Finally, the green view index was calculated according to the number of vegetation pixels in each photo (54). The average level of street green view index within the park's service area was used to reflect the quality of the natural environment around the park.

The park is influenced by the surrounding service functions. The richness of the surrounding functions is positively correlated with the park visitation rate (55). Parks will be transformed into ecological values due to the development of the park city concept, forming an integrated development of “industry-city.” The diversity of service functions in park's service area can provide convenience for visitors and promote the transformation of the park's ecological values into economic values. By analyzing cities' Point of Interest (POI) data, this study introduces the Shannon Wiener Diversity Index (SWDI) in order to quantify the diversity of service functions in park's service area. Species diversity can be measured using the Shannon Wiener Index, which has been utilized in urban research, such as evaluating street functional diversity (56). According to this study, the higher the Shannon Wiener index H, the greater the number of service functions available in the park's service area. A detailed overview of the formula is given below:


(1) $$S_{j}^{d}=-\sum {}_{j=1}^{m}P_{ji}\ln(P_{ji})$$

$S_{j}^{d}$ is the diversity of service functions in the service are of park *j*; *m* denotes the number of facilities in each category; *i* is the total number of facilities that fall within a specific classification; and *P*_*ji*_is the number of functional facilities in the service area of park *j*, which indicates the percentage of all facilities in the service area.

The attractiveness of park *j* is calculated using a weighted method based on the following formula:


(2) $$\begin{array}{l} S_{j}=\left[\gamma_{A}\frac{S_{j}^{a}}{max_{j\in J}S_{j}^{a}}\right]+\left[\gamma_{G}\frac{S_{j}^{g}}{max_{j\in J}S_{j}^{g}}\right]+\left[\gamma_{D\;}\frac{S_{j}^{d}}{max_{j\in J}S_{j}^{d}}\right]\forall j\in J \end{array}$$

γ_*A*_, γ_*G*_,γ_*D*_ reflect the impacts of the park area ($S_{j}^{a}$), the green view index in park *j*'s service area ($S_{j}^{g}$), and the diversity index of service function in park *j*'s service area ($S_{j}^{d}$) on the park attractiveness. Weight γ_*A*_+γ_*G*_+γ_*D*_ = 1. In this study, it is assumed that the weight of the three influencing factors is equal.

The park's attractiveness will influence the probability of people's subjective choice to visit the park. Luo (57) introduced the Huff model into the FCA method to capture the supply effect of capacity differences between available public sites. Xing (27) introduced the Huff model in 2SFCA to calculate the probability of population selection considering park size and qualities. Huff model (58) has been used many times in park-related studies, e.g., to measure the actual population attraction and service radius of green spaces. We introduced the Huff model and combined it with a Gaussian function (22) to calculate the selection probability of the population considering the parking area and the social and natural environment surrounding the park. The calculation formulas (3) and (4) are as follows.

Supply and demand ratio *R*_*j*_ of park *j* in the first stage is calculated using the 2SFCA method and the Huff model as follows:


(3) $$Prob_{i}=\frac{S_{j}t_{ij}G\left(t_{ij},t_{0}\right)}{\sum_{j\in \left\{t_{ij}\leq t_{0}\right\}}S_{j}t_{ij}G\left(t_{ij},t_{0}\right)}$$

(4) $$G\left(t_{ij},t_{0}\right)=\{\begin{array}{cc} \frac{e^{-\left(1/2\right)\times \left(t_{ij}/t_{0}\right)2}-e^{-\left(1/2\right)}}{1-e^{-\left(1/2\right)}},if,\; & t_{ij}\leq t_{0}\\ \;0,if,\; & t_{ij}>0 \end{array}$$

*Prob*_*i*_ is the probability of population selection based on the Huff model in *i* visiting park *j*; *t*_*ij*_ denotes the commuting time from *i* to *j*, and the Gaussian function (G) is the distance impedance coefficient; *S*_*j*_ is the attractiveness of park *j* and *t*_0_ is the psychological threshold associated with commute time.

The supply-demand ratio *R*_*j*_ of park *j* in the first stage is calculated using the 2SFCA method and the Huff model as follows:


(5) $$R_{j}=\frac{S_{j}}{\sum_{k\in \left\{t_{ij}\leq t_{0}\right\}}Prob_{ij}P_{i}G\left(t_{ij},t_{0}\right)}$$

*P*_*i*_ is the population at the position *i*, G is the time-distance friction, and *t*_0_ is the psychological threshold associated with commute time.

The supply-demand ratio *Rj* was summed up to estimate spatial accessibility to parks *A*_*i*_ and weighted by the distance decay coefficient *G* and the selection probability *Prob*_*ij*_ as follows:


(6) $$\begin{array}{l} A_{i}=\underset{i\in \left\{t_{ij}\leq t_{0}\right\}}{\sum }{Prob_{ij}R}_{j}G\left(t_{ij},t_{0}\right) \end{array}$$

### Equality index

To easily observe the supply and demand level of each residential unit in the whole region, we evaluate the equality of each residential unit to the park area by the formula (10, 59):


(7) $$\begin{array}{l} E_{i}=\frac{\max\left(R_{j}\right)}{\max\left(a_{i}\right)}\times \left(a_{i}\right) \end{array}$$

where, *R*_*j*_ is the supply-demand ratio and max(*R*_*j*_) is the maximum supply-demand ratio between the urban park and population demand; *a*_*i*_ is the accessibility value for each residential unit and max(*a*_*i*_) is the maximum accessibility value for the residential unit. The equality index *E*_*i*_ is classified into six classes that show the imbalance between population demand and park supply (25, 59) (Table 1).

**Table 1:** List of dependent variables.

**Class**	**Range of E_i_ value**	**Supply and demand status**	**Spatial equality**
I	*E*_*i*_ = 0	No supply	Serious inequality
II	0.25>*E*_*i*_>0	Very weak	Serious inequality
III	0.5>*E*_*i*_≥0.25	Weak	Relative inequality
IV	0.75>*E*_*i*_≥0.5	Good	Equality
V	1≥*E*_*i*_≥0.75	Very good	Relative equality
VI	*E*_*i*_>1	Oversupply	Serious inequality

### A single local moran's I

Local Indicators of Spatial Autoassociation (LISA) (60) were used to characterize the spatial agglomeration pattern of equality score in each residential grid unit with its neighboring residential unit. LISA is an effective tool for spatial autocorrelation analysis that can be used to identify the local association between an observation and its neighboring zones and to determine the presence of a statistically significant spatial cluster of variables (60, 61). The formula is as follows:


(8) $$\text{LocalMoran}\prime \text{sI}=\frac{n\left(\gamma_{i}-\bar{\gamma }\right)\sum_{j=1}^{m}W_{ij\left(\gamma_{i}-\bar{\gamma }\right)}}{\sum_{i=1}^{m}{\left(\gamma_{i}-\bar{\gamma }\right)}^{2}}$$

where, γ_*i*_ and γ_*j*_ are the equality scores of residential units *i* and *j*, $\bar{\gamma }$ is the mean value of the equality scores, *n* is the number of residential units, *m* is the number of residential units around residential unit *i*, and *W*_*ij*_ is the spatial weight matrix.

Spatial relationships between each residential grid unit with its neighboring residential unit can be categorized into five types: (a) high-high cluster (HH cluster), indicating high scores around grid residential units with high scores; (b) high-low cluster (HL cluster), indicating high scores around grid residential units with low scores; (c) low-high cluster (LH cluster), indicating high scores around grid residential unit with low scores; (d) low-low cluster (LL cluster), indicating low scores around grid residential unit with low scores; and (e) not significant cluster, indicating that spatial relationships are not significant.

### Lorentz curve and gini coefficient

**Figure 2:**
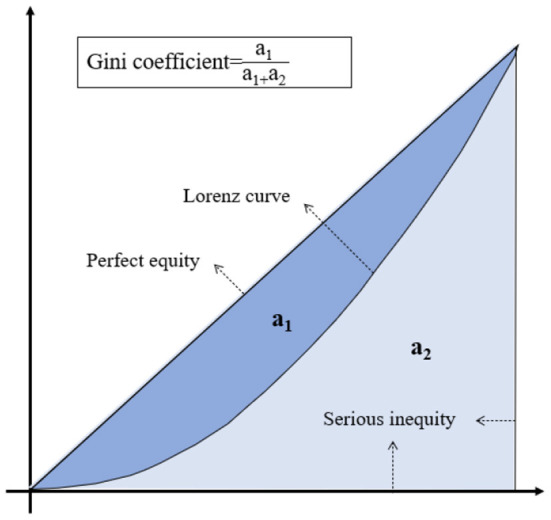
Lorenz curve and Gini coefficient conceptual chart.

The concentrated curve or Lorentz curve (62) is shown in Figure 2. It was originally proposed by Lorenz (63) to compare and analyze social income inequality. The Gini coefficient is determined from the Lorenz curve, and both are, respectively, used to measure the balance of certain resources visually and quantitatively. This approach has been applied in many scientific fields such as medical resources, education resources, green space resources, etc. The Gini coefficient is the ratio of the area between the absolute equality line and the Lorentz curve to the area between the absolute equality line and the absolute inequality line in the Lorentz graph (64).

In Figure 2, the deeper the Lorentz curve descends, the larger the area of *a*_1_ part will be, which indicates that the distribution of this resource will be more unbalanced, and the Gini coefficient will be relatively higher. The formula for calculating the Gini coefficient is as follows:


(9) $$G=1-\sum {}_{i-1}^{n}\left(P_{i}-P_{i-1}\right)\left(Q_{i}-Q_{i-1}\right)$$

*i* is the number of spatial statistical units, *P*_*i*_is the cumulative proportion of the population under the *i*^−*th*^ space unit, and *Q*_*i*_ is the cumulative proportion of park resources that are occupied in the *i*^−*th*^ spatial unit. The Gini coefficient varies in the range 0 ≤ G ≤ 1. A larger Gini coefficient indicates a less uniform distribution, whereas a smaller coefficient denotes greater uniformity (i.e., a more homogeneous distribution).

### Case study: Access to urban parks in Chengdu

#### Study area and research scale

**Figure 3:**
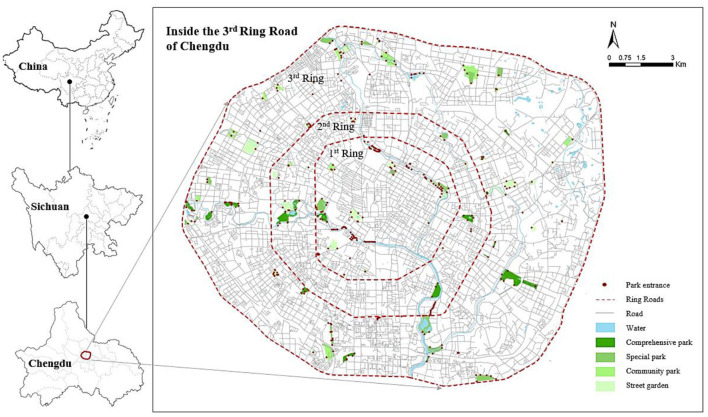
Location of the study area in Chengdu and spatial distribution of research subjects.

Chengdu is the capital of Sichuan province in southwest China with 21,192,000 inhabitants. With the development and expansion of the city, Chengdu gradually formed a concentric circular ring structure (1st ring, 2nd ring, 3rd ring), and residential areas gradually shifted from the core to the outside (65). As a study area, we selected the traffic loop within the 3rd ring road in Chengdu. This is the most mature and most characteristic central urban area of Chengdu with a total area of about 190 km^2^ (Figure 3).

The parks in this study are comprehensive parks, special parks, community parks and street gardens based on the Urban Green Space Classification Standard (CJJ/T85-2017), each with an area of more than 0.1 hectare. They are located within the third ring of Chengdu and are open to the public for free. The types of parks in this region are rich and comprehensive, long-term residents are less mobile, and there is also a marked contradiction between the growing demand of residents for recreation and the layout of parks.

We divided the study area into a 100 × 100 m grid for three reasons. First, in response to China's urban planning policy to start implementing grid-based community management, the study provides a practical reference value for it (14, 66). Second, previous studies used census block groups as units, and population demand points were geographic centroids or population-weighted centroids. Traditional studies usually use the geographic centroid of the census area to reflect the population distribution or achieve a uniform population distribution by using the area volume as the weight of the population distribution through the interpolation method. These methods hardly reflect the spatial distribution of the population in real life. Lee and Hong (67) divided Daegu into grids and discussed spatial differences from the perspective of geographic units. This facilitated a more intuitive interpretation of study results and diagnosing problem areas based on planning standards (67). A 100 × 100 m grid represents better resolution and can realize fine-scale population spatial distribution, reducing experimental error and providing support for smaller-scale equality research cases (14, 33).

#### Data sources and processing

**Figure 4:**
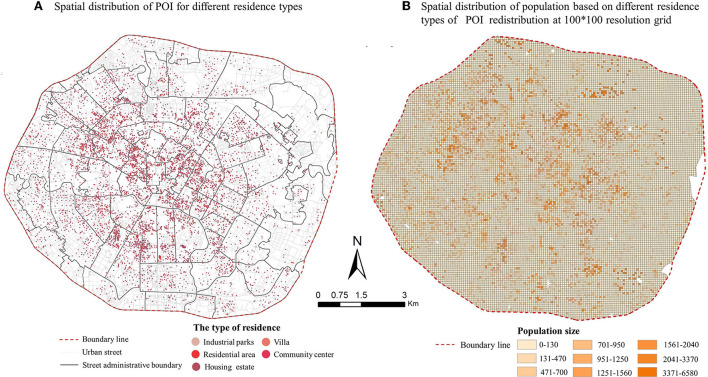
Spatial distribution of population in Chengdu, China. **(A)** Spatial distribution of POI for different residence types. **(B)** Spatial distribution of population based on different residence types of POI redistribution at 100 *times* 100 resolution grid.

Population data came from the Sixth National Census Key Data Bulletin and the Chengdu City Statistical Yearbook 2018. The study area was divided into 100 × 100 m grid using ArcGIS10.5, and the residential POI with the highest correlation with population distribution was used as an indicator of population redistribution to implement the secondary spatial distribution of the population in the study area (Figure 4).

The basic information and park entrance/exit data in the study area come from ShuiJingZhu map, Baidu Map, Chengdu Urban Green Line Control Atlas, and Chengdu Public open data platform. The traditional 2SFCA takes the centroid of the park as the supply point, but in fact people are considered to use the park when they arrive at the park entrance. Therefore, this study takes the park entrance as the supply point, which is more in line with reality. The service radii of urban parks are set according to the Urban Green Space Planning Standards (68).

Data on the road network in the study area were downloaded from OpenStreetMap (OSM). OSM not only provides up-to-date high-quality road network data for free, but also different types of roads, such as motorway, trunk, and footway. Based on different road types, we can also apply speed limits in our new O-D matrix calculation.

The scope of park services is usually determined by the type and size of the park. Referring to the goal of “300 meters to see green, 500 meters to see the park” proposed in the “Chengdu Park Urban Green Space System Planning (2019–2035)” (submitted for review), the service radius of the comprehensive park and special park (area >5,000 m^3^) is 500 m, and the service radius of the special park (area <5,000 m^3^), community park and street park is 300 m.

**Figure 5:**
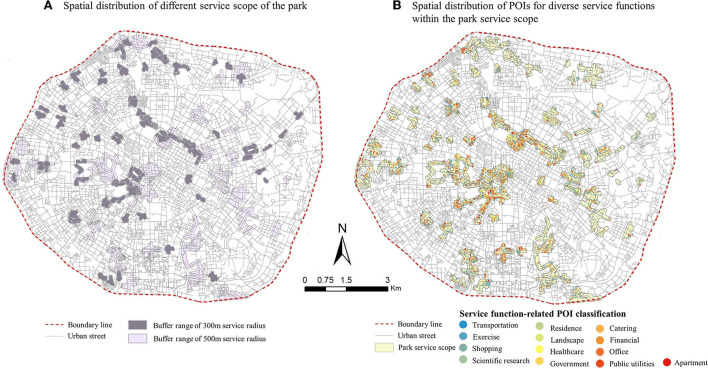
Screening out different types of service function POIs within the park's service area. **(A)** Spatial distribution of different service scope of the park. **(B)** Spatial distribution of POIs for diverse service functions within the park service scope.

In Figure 5, the 13 types of POIs used to calculate the diversity of service functions were obtained from Baidu Maps, which are scenic spots, catering services, public facilities services, transportation facilities services, companies and enterprises, shopping services, accommodation services, scientific, educational, and cultural services, government agencies and social organizations, healthcare, sports and leisure services, living services and financial services. ArcGIS was used to construct buffer zones along with the road network according to the service radius of the different parks, and all POI types within the service range were filtered. Then, SWDI was used to calculate the diversity of service functions of the park.

**Figure 6:**
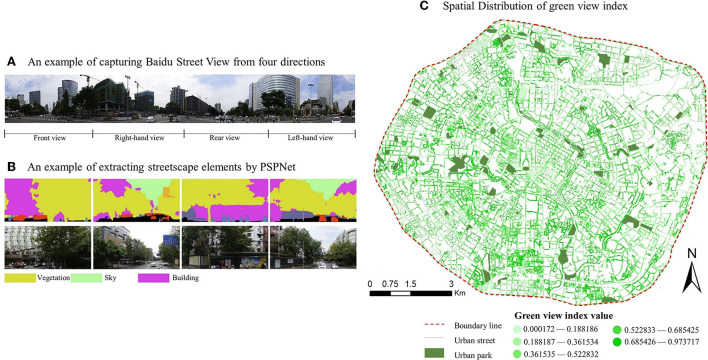
Obtaining method and spatial distribution of green view index.

Street view pictures (SVPs) for calculating the green view rate at a distance of 60 m from each road are first generated based on the OSM road network using ArcGIS platform. Application Programming Interface (API) of Baidu Maps is then called to obtain all the SVPs needed for each sampling point by setting parameters such as latitude, longitude, pitch angle, and yaw angle. In this study, each sampling point is based on the human view of the horizon. First, the pitch angle is set to 0°, and then the yaw angle is set to 0°, 90°, 180° and 270°, respectively, in order to obtain four images of the street scene in different directions. Then, the four images are put together into a panorama as shown in Figure 6. Pyramid Scene Parsing Network (PSPNet) is then used as a deep learning model to interpret the street view images into color groups, and then it was necessary to identify street elements including vegetation, sky and building. Each color represents a different street composition, and the proportion of the pixel size of each color in each photo represents the composition proportion of this element. The percentage of each component in the east, south, west and north direction of each sampling point is summed, and its average value is calculated in order to represent the average status of each component at the sampling point. In this study, the percentage of vegetation pixels in each image in the whole image was taken as the green view index, and then the average value of the four directions was calculated as the final green view index of the sampling point. Finally, the green view index was filtered using the same method as filtering POI points and the mean value was calculated.

**Figure 7:**
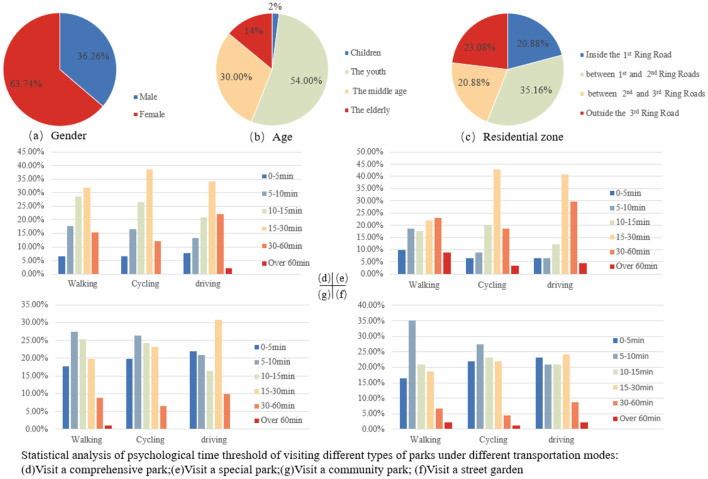
Statistical analysis of questionnaire results.

The time required to visit a park of different attractiveness varies from person to person and from time to time with different modes of transportation (69). In order to better understand people's actual commuting habits in their daily lives, this study conducted an online and offline questionnaire survey in September 2020 in the Chengdu metropolitan area at the same time. A total of 300 valid questionnaires were collected. The questionnaire (Appendix 1) included socioeconomic and demographic factors, the travel mode to visit different types of parks, as well as how much time they were willing to spend commuting this way (Figure 7). Limited by the questionnaire method, most of the respondents were middle-aged and young people, with fewer children and elderly people, and 63.74% of them were female.

The psychological time thresholds were obtained as follows. The first step was to calculate the average time to visit the 4 types of parks under 3 travel modes according to the statistical results of the questionnaire. By multiplying the middle value of each time period (60 min for over 60 min) by the percentage of that time period, and then summing the products of all time periods it is possible to obtain the average visit time for this type of park under this travel mode. To ensure that as many people as possible can reach the corresponding park, the maximum average time to visit the 4 types of parks under each travel mode was finally selected separately, and rounded to the nearest whole number to obtain the final psychological time threshold, i.e., the walking time threshold is set to 30 min, the cycling time threshold is set to 25 min, and the driving time threshold is set to 30 min. According to the average speed of daily transportation modes, this study sets the walking speed as 5 km/h, the cycling speed as 15 km/h, and the driving speed as 40 km/h.

## Results

### Analysis of distribution of spatial accessibility to parks

**Figure 8:**
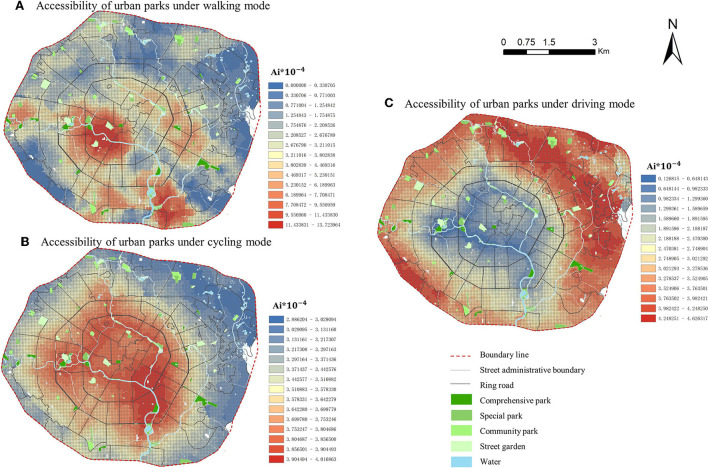
Spatial distribution of park accessibility under different travel modes in Chengdu, China.

In Figure 8, the spatial distribution of accessibility based on walking, cycling, and driving is very different. In the walking mode, due to the slow walking speed and the limited areas that can be reached within a certain time, the high accessibility areas are mainly grouped into clusters centered on parks. There are two core areas with high accessibility in the study area. One core area is located on the west side of the 1st Ring Road and the other sub-core area is located on the south side between the 2nd and 3rd Ring Roads. The area with low overall accessibility was significantly larger than the area with high overall accessibility.

In the cycling mode, the spatial distribution of accessibility is circular, with the highest accessibility along the Fu and Nan rivers within the 1st Ring Road and gradually decreasing toward the periphery, while the lowest accessibility is on the eastern side between the 2nd and 3rd Ring Roads.

In the driving mode, the spatial distribution of accessibility is similar to that of cycling, which is also a circular plane distribution. However, the distribution of high and low accessibility areas is completely opposite to that of cycling. Driving accessibility is lowest along Fu and Nan rivers and gradually increases toward the periphery, with the highest accessibility mainly on the east side between the 2nd and 3rd rings. The influence of the Fu River and the Nan River on the development of urban transport is visible in cycling and driving. With the promotion of green travel and the return of roads to the people, the slow riverside system has gradually become a public place for leisure and fitness, so the cycling accessibility along the riverside is high, while driving accessibility is low.

### Statistical analysis of spatial accessibility to parks

Spatial disparities in park accessibility were different between ring roads under different travel modes. The average level of accessibility is as follows: driving > walking > cycling. In Table 2, in driving mode, park accessibility is relatively equal, with an average accessibility value of 3.53^*^10^−4^ m^2^/person and a standard deviation of <1 m^2^/person. This indicates that rapid transportation provides relatively balanced opportunities for residents to visit the park in the study area. In walking mode, park accessibility varies greatly from region to region, and the relationship of accessibility is as follows: inside 1st Ring Road > between 2nd and 3rd Ring Roads > between 1st and 2nd Ring Roads. The largest difference is within 1st Ring Road, with a maximum accessibility value of 13.816^*^10^−4^ m^2^/person and a minimum value of 0 m^2^/person. This indicates that while it is easy for some residents to walk to the park, it is relatively difficult for some residents to do so. In cycling mode, park accessibility is relatively stable and balanced in each region, with the standard deviation <1 m^2^/person. The relationship of accessibility is as follows: between 2nd and 3rd Ring Roads > between 1st and 2nd Ring Roads > inside 1st Ring Road. The accessibility of the area outside the 2nd Ring Road is lower than inside the 2nd Ring Road, which indicates that it is more convenient for residents living inside the 2nd Ring Road to cycle to the park than those living outside the 2nd Ring Road.

**Table 2:** Statistical analysis of park accessibility in different regions of Chengdu, China.

**Traffic mode**	**Zones**	**Min** (*10^−4^)	**Max** (*10^−4^)	**Mean** (*10^−4^)	**Standard deviation**	**Underserved area (%)**
Walking	ALL	0	13.816	3.057	2.617	4.41%
	Inside 1st Ring Road	0	13.816	5.550	3.376	0.098%
	Between 1st and 2nd Ring Roads	0	12.200	3.780	1.850	0.082%
	Between 2nd and 3rd Ring Roads	0	12.982	2.358	2.167	6.265%
Cycling	All	0	4.630	2.514	1.212	2.200%
	Inside 1st Ring Road	0	4.630	3.980	0.273	0.098%
	Between 1st and 2nd Ring Roads	0	4.626	3.659	0.456	0.082%
	Between 2nd and 3rd Ring Roads	0	4.319	1.949	0.983	3.098%
Driving	ALL	0	4.017	3.529	0.590	2.200%
	Inside 1st Ring Road	0	3.525	3.201	0.163	0.098%
	Between 1st and 2nd Ring Roads	0	3.896	3.401	0.211	0.082%
	Between 2nd and 3rd Ring Roads	0	4.017	3.629	0.667	1.832%

### Analysis of equality in access to parks

**Figure 9:**
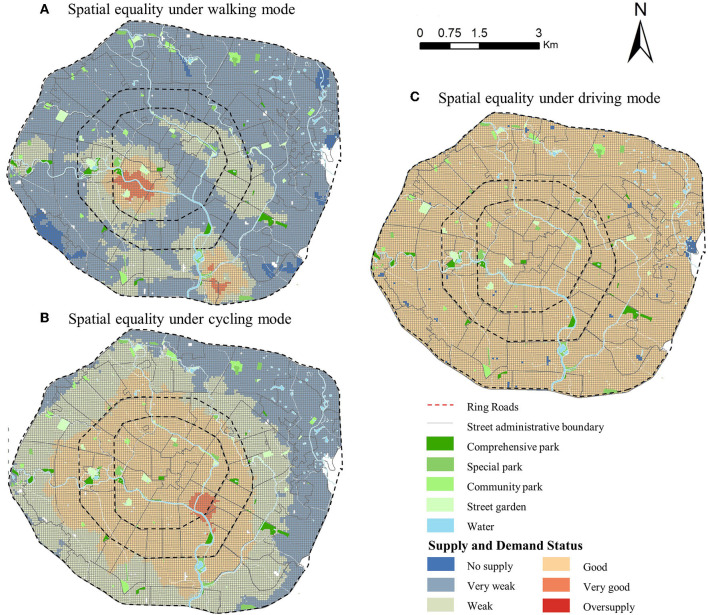
Spatial equality distribution under different travel modes in Chengdu, China.

**Figure 10:**
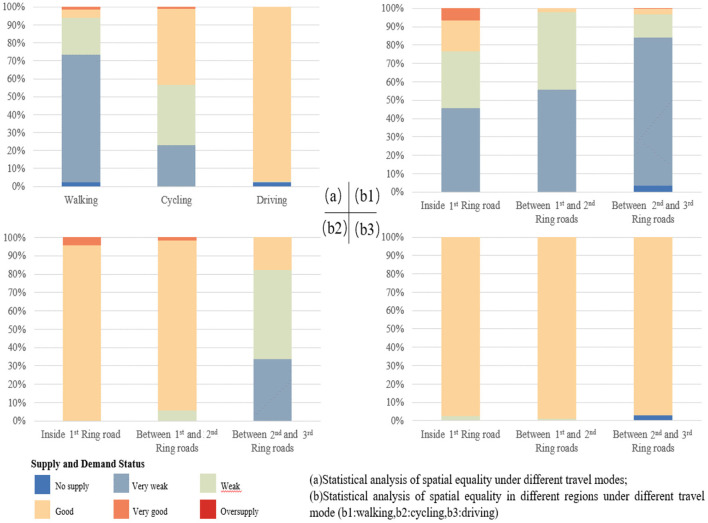
Statistical analysis of Spatial equality distribution under different travel modes in Chengdu, China.

As shown in Figure 9, there are significant differences in the spatial distribution of supply and demand for the three travel modes. There are no areas of oversupply under the three modes of travel, but there are extremely inequitable areas with no supply. The overall spatial equality inside the 2nd Ring Road is better than outside the 2nd Ring Road. Due to the earlier construction of the city center, the 2nd Ring Road not only contains many large parks with long history, but also has mature infrastructure and more permanent residents. The whole region has the best supply-demand relationship in the driving mode. In Figure 10(a), almost the whole region is in a state of good supply equality, followed by cycling with 43.59% of the region in a state of equality. The worst is the walking mode with 93.85% of the region in a state of inequality. This indicates that the change in the travel mode has a significant effect on the spatial equality of the parks. The faster the travel mode in the same travel time range, the better the spatial equality.

In the walking mode, 71.3% of the area has a very weak supply with serious inequality. The proportion of very weak supply gradually increases from the 1st to the 3rd Ring Road, with spatial equality becoming increasingly unequal. The types of supply and demand within the 1st Ring Road are the richest, indicating a significant gap in spatial equality. The spatial equality of the south side of the study area is better than the north side, because the north side is part of the old redevelopment area, while the south side is the currently fastest developing area of Chengdu and therefore has a better overall level. Influenced by the small scale of walking trips, the study of its spatial equality is more suitable for fine-grained small-scale areas in order to reflect the subtle inequitable areas in urban construction.

In Figure 10(b2), in the cycling mode, the supply and demand status within the 2nd Ring Road are good and are in the state of equality. Among them, the area located on the southeast side of the 1st Ring Road has a very good supply with a total of 10%. The area within the 2nd Ring Road is more mature and with the construction of greenways at all levels is more convenient to cycle to the park when traffic is congested. Only 33.08% of the area between the 2nd and 3rd Ring Roads on the eastern side of the city is very poorly supplied. This area is the new eastern part of Chengdu, which is still in the early stage of development, with few parks and a lack of cycling conditions, so the spatial equality between walking and cycling is very weak.

In Figure 10(b3), in the driving mode, since the road network in the city center is well-established, the choice of driving is less hindered by time. As long as residents want to visit the park, the opportunity to visit each park is relatively equitable, and the supply and demand for the park are mainly influenced by personal choices.

### Spatial autocorrelation analysis of park accessibility

**Figure 11:**
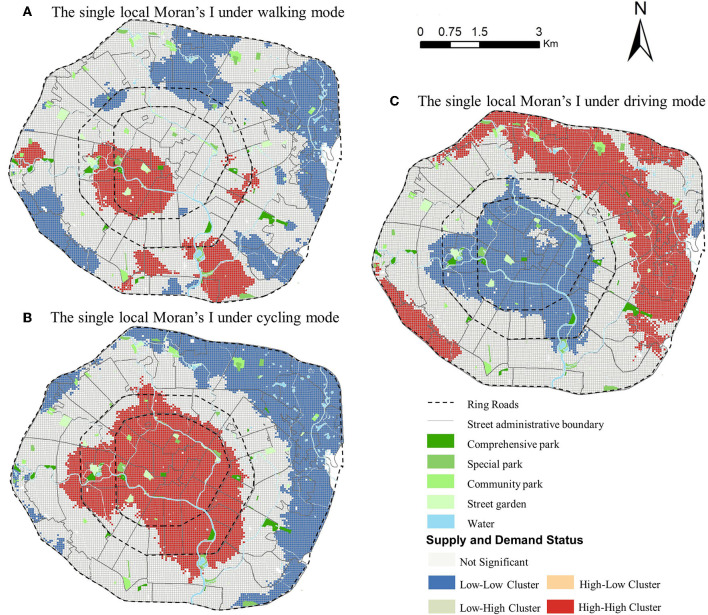
Spatial autocorrelation analysis under different travel modes in Chengdu, China.

In Figure 11, an examination of agglomeration data shows an apparent spatial disparity in the equality in park accessibility in different districts. The cluster types within the three modes of transportation are mainly HH&LL clusters and not significant clusters. The HL&LH cluster also exists in several residential units in walking and cycling mode.

In the walking mode, there are two significant areas of the HH cluster located on the west side of the 2nd Ring Road and the south side between the 2nd Ring Road and the 3rd Ring Road. This indicates that the residents of these two areas enjoy higher and equal access to the park. The area of the LL cluster in walking mode is about twice as large as that of the HH cluster. There are also several communities with HL&LH clusters and these residents have to overcome the problem of distance from the park.

In the cycling mode, the HH cluster and the LL cluster are similar in size. However, the HH cluster areas are located in the downtown area, while LL cluster areas are mainly located in the east near the edge of the 3rd Ring Road This indicates that those who live in the downtown area enjoy a higher level of equal access to the park than those living in the 3rd Ring Road.

In the driving mode, the area of the HH cluster is slightly larger than that of the LL cluster. Its spatial distribution is just the opposite in the cycling mode, indicating that the traffic in the 3rd Ring Road is smoother than that in the 2nd Ring Road, and it is convenient to reach and enjoy the park.

### Equality analysis of the park resources

**Figure 12:**
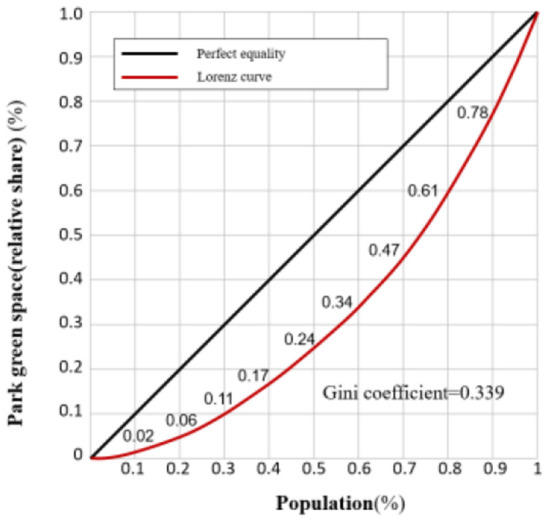
Lorenz curve diagram.

In this study, the Lorenz curve and the Gini coefficient are used to estimate the balance between park resources and population within the 3rd Ring Road in Chengdu. There is still a certain imbalance between the supply and demand of parks and citizens within the 3rd Ring Road, i.e., some citizens enjoy fewer park resources, with 10% of the citizens' demand being matched by only 2% of the park's resources providing supply services, and 20% of the citizens' demand being matched by only 6% of the park's resources providing services. Some citizens enjoy relatively more park resources, with 10% of the demand of this group of citizens enjoying 21.72% of the supply services provided by the park resources, while 20% of the citizens are matched with 38.77% of the supply services provided by the park resources (Figure 12). The Gini coefficient of the allocation of park resources enjoyed by different population proportions in the 3rd Ring Road is 0.339. According to the relevant organizations of the United Nations, the Gini coefficient is divided into five parts, as detailed in Table 2. In Table 3, the distribution of park supply and demand in the study area is in a relatively reasonable intermediate state, not exceeding the warning line of 0.4, but there is a certain gap from reaching equilibrium.

**Table 3:** Gini coefficient segmentation table showing equality in resources.

**Gini coefficient segmentation**	**Meaning**
<0.2	Absolute equality
0.2–0.3	Equality
0.3–0.4	Relatively equality
0.4–0.5	Low inequality
>0.5	High inequality

## Discussion

### Improvement of the 2SFCA method based on big data and machine learning

Growing evidence of the positive relationship between park accessibility and human health and wellbeing has prompted calls for a better understanding of the equality of park accessibility (70, 71). From the perspective of overall urban planning, this study attempts to consider the positive intervention of the surrounding urban environment on the accessibility of the park. Based on user needs, it improves the 2SFCA method based on big data and machine learning, and constructs a new evaluation system, making the following contributions to the research field:

(1) The study provides evidence for fine-scale research. Although there have been many studies on the spatial equality of parks (28, 72), evidence for the access to urban parks based on a supply-demand balance at grid resolution is lacking (14). Using a refined 100^*^100 m grid resolution as a base unit can help more comprehensively diagnose finer differences in supply and demand within a region, which facilitates more focused identification of inequitable areas for targeted optimization. It also provides a practical reference for China's attempts to perform grid-based management of urban communities.
(2) This study used big data and machine learning to improve evaluation efficiency and accuracy. Using residential POI for population redistribution can reflect a more realistic spatial distribution of population. Through a machine learning method, Baidu Street View images and POI data reflecting the functions of urban services are used to measure the urban environment around the park on a large scale and realize spatial distribution visualization. The use of multi-city big data and new computer technologies can achieve accurate and large-scale measurements, which can reduce evaluation error and improve efficiency.
(3) This study improves the applicability of research from a human-centered perspective. The traditional GIS-based approach has been heavily criticized for ignoring people's preferences as measured through real movement and usage (35). This study considers the reality that people will subjectively choose to visit different parks due to the influence of park attractiveness and personal preferences and introduces the Huff model to estimate the probability that people will choose to visit each park. The cost of commuting time that people are willing to spend visiting different parks by different transportation modes depends largely on whether people are willing to overcome major barriers to visit parks. Therefore, this study determines the time threshold by directly investigating and interviewing users, making the study closer to people's daily life.

### The comprehensive analysis from both spatial equality and quantitative equality

Unlike urban planners who are accustomed to using statistical indicators to evaluate the overall level of urban park construction, this study provides a method to evaluate park equality in two dimensions: spatial equality and quantitative equality. In case the overall number of parks is sufficient, residents distributed in different areas do not necessarily have equal access to urban parks. By revealing differences in the distribution of park space equality in the case of overall park equality at a refined scale, we can highlight key areas that may need to be optimized in park layout and inform planners to make more informed and accurate decisions. This is a method that can quickly and directly quantify and visualize the equality of park resources enjoyed by urban residents from an urban scale and can be better applied in practice. However, we do not recommend abandoning the indicators used in the past to evaluate overall construction of the park. These two measurement methods can complement each other, not replace each other.

According to the Gini coefficient of 0.339, the overall supply of park resources in the 3rd Ring Road of Chengdu is sufficient to meet the needs of residents. However, there are significant differences in the spatial distribution of park access equality under different travel modes. People do not enjoy equal opportunities to enter the park. The oversupply of park resources in some areas causes waste, and the insufficient supply needs to be urgently optimized in some areas. The walking mode should focus on optimizing the quality and quantity of parks according to population density. In areas where accessibility is 0, there is still a need to consider adding a new park. Secondly, in a place with limited conditions for construction, it is necessary to make full use of the green streets as new activity places. Cycling to the park in the area between the 2nd and 3rd Ring Roads is almost unequal. In undeveloped areas near the 3rd Ring Road, new parks should be planned, and the walking and cycling network should be improved. Due to the limited construction conditions at the 2nd Ring Road, the enrichment of service functions around the park can be considered to attract more people to actively break the physical space barrier to visit less-used park, promote population flow, and dynamically adjust the balance of park utilization rates. Although almost the entire area of equality in access to urban parks is in the driving mode, from a social equality perspective, whether or not to own a car is originally a reflection of the gap between the rich and the poor in society. Urban households that do not have a car have a greater disadvantage, so it is impossible to advocate that everyone can travel by car, but public transportation can be continuously optimized.

### Future applicability and limitations

This study provides a new perspective on alleviating inequalities in access to urban parks and the allocation of public resources in high-density cities. For mega-city centers where building land is scarce, acquiring land for new parks is the most expensive and complex measure to address the inequality of park accessibility (71). Addressing user needs and subjective preferences in order to maximize the quality of existing park construction and thereby maximize the efficiency of the use of existing parks is relatively achievable. Parks are only part of the city, and urban public resources complement each other to help maximize service benefits. People visit parks to enjoy the ecosystem services they provide, and if other urban public resources around the parks could replace some of the services that parks provide, this would alleviate the inequality created by the lack of park services (11). This study also provides other cities with a comprehensive method of assessing equality in access to urban parks on a large scale, which is useful in helping urban planners identify key locations for optimizing park distribution and providing scientific support for park planning.

Several limitations of this study should be acknowledged. First, the uncertainty is caused by the different grid scales. Different resolutions have different grid masses, and redistribution of resident data to partitions can produce errors (14). Potential uncertainties or biases due to different grid resolutions are not discussed in this study. Future research can investigate the impact of different research scales on the accessibility results. Second, the effect of population stratification on the equality of park accessibility was not analyzed. Studies have shown that people of different economic statuses and races have different access to parks (71, 73). Other researchers have found that vulnerable groups are not treated unequally (74). All these studies indicate that the impact of population background on park accessibility is not yet clear, and further studies on the equality of park accessibility can be combined with population information. Third, the factors influencing the attractiveness of the park are not considered comprehensively. Due to many research objects in this study, the quality of the park's internal construction was not comprehensively evaluated. In addition, this study did not consider the weight of different factors influencing park attractiveness. Future research should further increase the number of influencing factors for park attractiveness evaluation and explore the specific influence weights of each factor to improve the accuracy of the evaluation model. Fourth, the mode of travel is not considered thoroughly enough. This study does not consider the public transport modes such as metro and bus and the fact that people usually combine different travel modes in their daily life. Future related studies may consider multiple composite transportation modes. Finally, due to the limited length of the article, this study only analyzed the differences in spatial accessibility under different travel modes and did not further compare and analyze the spatial accessibility of different types of parks. Future research can further cross-analyze the accessibility distribution rules of different park types under different travel modes. In future research, researchers can also consider introducing new technologies such as big data, artificial intelligence, and virtual experiments, and always focus on human needs to develop new research methods, which will provide more opportunities to study the layout of urban parks.

## Conclusion

This study assesses equality in access to urban parks under different travel modes through the supply-demand adjusted 2SFCA method. This is important for improving the efficiency of park ecosystem services and optimizing park layouts from an urban planning perspective. Under the positive intervention of the urban environment around the park, Chengdu's evaluation results show that the overall amount of park resources is in a relatively reasonable intermediate state, but the spatial distribution of park accessibility is still unequal. The layout of spatial accessibility in the walking mode is distributed in the form of kernel density aggregation, with a smaller radius, which is more influenced by the high-grade park. The spatial distribution of cycling and driving accessibility is circular and closely related to urban rivers, while the trend of accessibility distribution is opposite. Inequality in spatial accessibility is greatest for walking to each park compared to cycling and driving and shows that convenient transportation can alleviate inequality in access to parks. We propose to focus on the construction of green streets and waterfront spaces, the enrichment of services around parks, and the speed of public transportation, so as to alleviate the inequality in access to urban parks. A method of measuring access based on the coordinated development of peri-urban resources would prove to be an effective tool for equality-oriented urban planners to identify and narrow the various evident disparities in public facilities.

## Data availability statement

The original contributions presented in the study are included in the article/supplementary material, further inquiries can be directed to the corresponding author.

## Author contributions

WD and SN participated in the study concept and design. DP collected the data. WD, SY, and YL participated in the analysis and interpretation of the data. WD and SN critically revised the manuscript. All authors contributed to the article and approved the submitted version.

## Funding

This research was funded by the China Postdoctoral Science Foundation (No. 2020M673222) and the National Natural Science Foundation of China (No. 52108021).

## Conflict of interest

Authors WD and DP are employed by China Southwest Geotechnical Investigation and Design Institute Co., Ltd., China. The remaining authors declare that the research was conducted in the absence of any commercial or financial relationships that could be construed as a potential conflict of interest.

## Publisher's note

All claims expressed in this article are solely those of the authors and do not necessarily represent those of their affiliated organizations, or those of the publisher, the editors and the reviewers. Any product that may be evaluated in this article, or claim that may be made by its manufacturer, is not guaranteed or endorsed by the publisher.
