# Supplementary material for: Measuring equality in access to urban parks: A big data analysis from Chengdu
Source: Front Public Health. 2022 Oct 6;10:1022666. doi: 10.3389/fpubh.2022.1022666 (PMC9590313; doi:10.3389/fpubh.2022.1022666)
Supplement: Supplementary file 1 [file Data_Sheet_1.docx]

**Appendix.1**

**Questionnaire on the accessibility of urban parks within 3^rd^ Ring Road of Chengdu**

**1. Gender**(Single choice)**:** 🞎M 🞎F

**2.Age**(Single choice)**:**

🞎Children(<18)

🞎The youth(18-35)

🞎The middle age(35-60)

🞎The elderly(>60)

**3.Residential zone**(Single choice)**:**

🞎Inside the 1^st^ Ring Road

🞎Between 1^st^ and 2^nd^ Ring Roads

🞎between 2^nd^ and 3^rd^ Ring Roads

🞎Outside the 3^rd^ Ring Road

**4.If you want to go to a comprehensive park, which mode of travel would you prefer? How much commuting time are you willing to spend in this mode of travel?**(Multiple choice)

| Travel modes modes  Time | 0-5 minutes | 5-10 minutes | 10-15 minutes | 15-30 minutes | 30-60 minutes | 60 minutes or more |
| --- | --- | --- | --- | --- | --- | --- |
| Walking | 🞎 | 🞎 | 🞎 | 🞎 | 🞎 | 🞎 |
| Cycling | 🞎 | 🞎 | 🞎 | 🞎 | 🞎 | 🞎 |
| Driving | 🞎 | 🞎 | 🞎 | 🞎 | 🞎 | 🞎 |

**5.If you want to go to a special park, which mode of travel would you prefer? How much commuting time are you willing to spend in this mode of travel?**

(Multiple choice)

| Travel modes modes  Time | 0-5 minutes | 5-10 minutes | 10-15 minutes | 15-30 minutes | 30-60 minutes | 60 minutes or more |
| --- | --- | --- | --- | --- | --- | --- |
| Walking | 🞎 | 🞎 | 🞎 | 🞎 | 🞎 | 🞎 |
| Cycling | 🞎 | 🞎 | 🞎 | 🞎 | 🞎 | 🞎 |
| Driving | 🞎 | 🞎 | 🞎 | 🞎 | 🞎 | 🞎 |

**6.If you want to go to a community park, which mode of travel would you prefer? How much commuting time are you willing to spend in this mode of travel?**

(Multiple choice)

| Travel modes modes  Time | 0-5 minutes | 5-10 minutes | 10-15 minutes | 15-30 minutes | 30-60 minutes | 60 minutes or more |
| --- | --- | --- | --- | --- | --- | --- |
| Walking | 🞎 | 🞎 | 🞎 | 🞎 | 🞎 | 🞎 |
| Cycling | 🞎 | 🞎 | 🞎 | 🞎 | 🞎 | 🞎 |
| Driving | 🞎 | 🞎 | 🞎 | 🞎 | 🞎 | 🞎 |

**7.If you want to go to a street garden, which mode of travel would you prefer? How much commuting time are you willing to spend in this mode of travel?**

(Multiple choice)

| Travel modes modes  Time | 0-5 minutes | 5-10 minutes | 10-15 minutes | 15-30 minutes | 30-60 minutes | 60 minutes or more |
| --- | --- | --- | --- | --- | --- | --- |
| Walking | 🞎 | 🞎 | 🞎 | 🞎 | 🞎 | 🞎 |
| Cycling | 🞎 | 🞎 | 🞎 | 🞎 | 🞎 | 🞎 |
| Driving | 🞎 | 🞎 | 🞎 | 🞎 | 🞎 | 🞎 |

附录一

| 街道 | 区 | 总人口 | 总人口分性别 | | 总人口分年龄 | | |
| --- | --- | --- | --- | --- | --- | --- | --- |
|  |  |  | 总人口男 | 总人口女 | 0-14岁 | 15-64岁 | 65岁及以上 |
| 圣灯街道 | 成华区 | 16600 | 8415 | 8185 | 2110 | 13257 | 1233 |
| 猛追湾街道 | 成华区 | 70107 | 34960 | 35147 | 4987 | 55023 | 10097 |
| 跳蹬河街道 | 成华区 | 37738 | 19225 | 18513 | 3626 | 29733 | 4379 |
| 保和街道 | 成华区 | 50286 | 27403 | 22883 | 6131 | 41453 | 2702 |
| 二仙桥街道 | 成华区 | 57872 | 32078 | 25794 | 3635 | 49479 | 4758 |
| 双水碾街道 | 成华区 | 60352 | 30892 | 29460 | 7406 | 49348 | 3598 |
| 建设路街道 | 成华区 | 63956 | 33172 | 30784 | 4974 | 50610 | 8372 |
| 龙潭街道 | 成华区 | 67778 | 34966 | 32812 | 9066 | 53296 | 5416 |
| 青龙街道 | 成华区 | 164479 | 86618 | 77861 | 21072 | 134441 | 8966 |
| 桃蹊路街道 | 成华区 | 89107 | 45061 | 44046 | 10471 | 70253 | 8383 |
| 新鸿路街道 | 成华区 | 61668 | 30830 | 30838 | 4846 | 48901 | 7921 |
| 万年场街道 | 成华区 | 76467 | 39553 | 36914 | 7968 | 62479 | 6020 |
| 双桥子街道 | 成华区 | 77541 | 38072 | 39469 | 5980 | 59033 | 12528 |
| 府青路街道 | 成华区 | 44834 | 22504 | 22330 | 4463 | 35438 | 4933 |
| 凤凰山街道 | 金牛区 | 4974 | 2548 | 2426 | 827 | 3895 | 252 |
| 西安路街道 | 金牛区 | 87021 | 42229 | 44792 | 6570 | 69771 | 10680 |
| 茶店子街道 | 金牛区 | 92181 | 45418 | 46763 | 9690 | 73824 | 8667 |
| 五块石街道 | 金牛区 | 51408 | 26487 | 24921 | 6200 | 42693 | 2515 |
| 西华街道 | 金牛区 | 111596 | 63433 | 48163 | 9352 | 98566 | 3678 |
| 驷马桥街道 | 金牛区 | 116239 | 59179 | 57060 | 10134 | 93238 | 12867 |
| 九里堤街道 | 金牛区 | 68502 | 35633 | 32869 | 6968 | 56423 | 5111 |
| 营门口街道 | 金牛区 | 90861 | 48129 | 42732 | 9891 | 76095 | 4875 |
| 荷花池街道 | 金牛区 | 62742 | 31948 | 30794 | 5607 | 49632 | 7503 |
| 人民北路街道 | 金牛区 | 77097 | 37823 | 39274 | 6396 | 59470 | 11231 |
| 抚琴街道 | 金牛区 | 106754 | 52556 | 54198 | 8197 | 86387 | 12170 |
| 黄忠街道 | 金牛区 | 39171 | 19030 | 20141 | 4290 | 31319 | 3562 |
| 金泉街道 | 金牛区 | 83343 | 43154 | 40189 | 10296 | 68552 | 4495 |
| 沙河源街道 | 金牛区 | 123727 | 66029 | 57698 | 16604 | 101563 | 5560 |
| 光华街道 | 青羊区 | 79765 | 37920 | 41845 | 7579 | 65606 | 6580 |
| 金沙街道 | 青羊区 | 31100 | 16135 | 14965 | 3499 | 25592 | 2009 |
| 草市街街道 | 青羊区 | 41221 | 20222 | 20999 | 2663 | 34321 | 4237 |
| 西御河街道 | 青羊区 | 32568 | 15197 | 17371 | 2490 | 25835 | 4243 |
| 新华西路街道 | 青羊区 | 53711 | 26472 | 27239 | 2821 | 47593 | 3297 |
| 草堂街道 | 青羊区 | 50295 | 24153 | 26142 | 4040 | 38928 | 7327 |
| 苏坡街道 | 青羊区 | 74268 | 39297 | 34971 | 8660 | 61691 | 3917 |
| 少城街道 | 青羊区 | 58837 | 28055 | 30782 | 5319 | 45920 | 7598 |
| 府南街道 | 青羊区 | 105005 | 51051 | 53954 | 10571 | 83864 | 10570 |
| 东坡街道 | 青羊区 | 55421 | 26746 | 28675 | 7735 | 43743 | 3943 |
| 汪家拐街道 | 青羊区 | 58911 | 28285 | 30626 | 4308 | 46449 | 8154 |
| 太升路街道 | 青羊区 | 58834 | 28860 | 29974 | 4227 | 47506 | 7101 |
| 机投桥街道 | 武侯区 | 103297 | 55347 | 47950 | 13311 | 86109 | 3877 |
| 华兴街道 | 武侯区 | 47154 | 25854 | 21300 | 4828 | 40845 | 1481 |
| 晋阳街道 | 武侯区 | 75440 | 37559 | 37881 | 9223 | 59583 | 6634 |
| 望江路街道 | 武侯区 | 92647 | 45954 | 46693 | 6483 | 77238 | 8926 |
| 双楠街道 | 武侯区 | 97700 | 49531 | 48169 | 9528 | 78732 | 9440 |
| 簇锦街道 | 武侯区 | 77647 | 41993 | 35654 | 8475 | 65484 | 3688 |
| 浆洗街街道 | 武侯区 | 62804 | 31841 | 30963 | 4522 | 51486 | 6796 |
| 玉林街道 | 武侯区 | 80734 | 39159 | 41575 | 6539 | 63730 | 10465 |
| 肖家河街道 | 武侯区 | 46827 | 23866 | 22961 | 4024 | 38790 | 4013 |
| 石羊场街道 | 武侯区 | 85196 | 45326 | 39870 | 9051 | 71138 | 5007 |
| 火车南站街道 | 武侯区 | 72386 | 35789 | 36597 | 7171 | 60037 | 5178 |
| 跳伞塔街道 | 武侯区 | 69153 | 33682 | 35471 | 5955 | 55412 | 7786 |
| 芳草街道 | 武侯区 | 96777 | 46651 | 50126 | 9842 | 76807 | 10128 |
| 桂溪街道 | 武侯区 | 63096 | 35632 | 27464 | 6173 | 53588 | 3335 |
| 红牌楼街道 | 武侯区 | 105770 | 53545 | 52225 | 12084 | 87360 | 6326 |
| 督院街街道 | 锦江区 | 25687 | 12825 | 12862 | 2443 | 20247 | 2997 |
| 盐市口街道 | 锦江区 | 11211 | 5764 | 5447 | 675 | 9342 | 1194 |
| 柳江街道 | 锦江区 | 52247 | 27711 | 24536 | 5423 | 43698 | 3126 |
| 水井坊街道 | 锦江区 | 27265 | 13756 | 13509 | 1972 | 21718 | 3575 |
| 书院街街道 | 锦江区 | 48078 | 23607 | 24471 | 3849 | 38111 | 6118 |
| 合江亭街道 | 锦江区 | 45090 | 22507 | 22583 | 3031 | 36850 | 5209 |
| 龙舟路街道 | 锦江区 | 45260 | 23233 | 22027 | 3852 | 36646 | 4762 |
| 沙河街道 | 锦江区 | 13710 | 7331 | 6379 | 1139 | 11136 | 1435 |
| 牛市口街道 | 锦江区 | 46741 | 24060 | 22681 | 3587 | 37410 | 5744 |
| 春熙路街道 | 锦江区 | 22216 | 10857 | 11359 | 1434 | 17947 | 2835 |
| 莲新街道 | 锦江区 | 59870 | 29501 | 30369 | 5754 | 48285 | 5831 |
| 成龙路街道 | 锦江区 | 113750 | 56710 | 57040 | 12938 | 92725 | 8087 |
| 狮子山街道 | 锦江区 | 64826 | 28164 | 36662 | 3395 | 57983 | 3448 |
| 双桂路街道 | 锦江区 | 24384 | 13839 | 10545 | 1944 | 19999 | 2441 |
| 东光街道 | 锦江区 | 65616 | 32461 | 33155 | 7235 | 52085 | 6296 |
